# Silencing of functional p53 attenuates NAFLD by promoting HMGB1-related autophagy induction

**DOI:** 10.1007/s12072-020-10068-4

**Published:** 2020-06-30

**Authors:** Xuequn Zhang, Yiming Lin, Sisi Lin, Chunxiao Li, Jianguo Gao, Zemin Feng, Jinghua Wang, Jie Zhang, Hong Zhang, Yuwei Zhang, Xueyang Chen, Shenghui Chen, Chengfu Xu, Youming Li, Chaohui Yu, Hang Zeng

**Affiliations:** 1 Department of GastroenterologyFirst Affiliated HospitalSchool of Medicine, Zhejiang University, Hangzhou, 310003 China; 2 Department of Pharmacy, Zhejiang Provincial People’s Hospital, Hangzhou, 310014 China

**Keywords:** p53, Nonalcoholic fatty liver disease, Autophagy, High mobility group box 1, p62, High-fat diet, Mice, HepG2, Huh7, Mouse primary hepatocytes

## Abstract

**Background and aim:**

Nonalcoholic fatty liver disease (NAFLD) is a common chronic liver disease worldwide, but its pathogenesis remains imprecisely understood and requires further clarification. Recently, the tumor suppressor p53 has received growing attention for its role in metabolic diseases. In this study, we performed in vivo and in vitro experiments to identify the contribution of p53–autophagy regulation to NAFLD.

**Methods:**

Livers from wild-type and p53 knockout mice as well as p53-functional HepG2 cells and p53-dysfunctional Huh7 cells were examined for autophagy status and HMGB1 translocation. In vivo and in vitro NAFLD models were established, and steatosis was detected. In the cell models, autophagy status and steatosis were examined by p53 and/or HMGB1 silencing.

**Results:**

First, the silencing of p53 could induce autophagy both in vivo and in vitro. In addition, p53 knockout attenuated high-fat diet-induced NAFLD in mice. Similarly, knockdown of p53 could alleviate palmitate-induced lipid accumulation in cell models. Furthermore, high mobility group box 1 (HMGB1) was proven to contribute to the effect of silencing p53 on alleviating NAFLD in vitro as an autophagy regulator.

**Conclusion:**

The anti-NAFLD effect of functional p53 silencing is associated with the HMGB1-mediated induction of autophagy.

**Graphic abstract:**

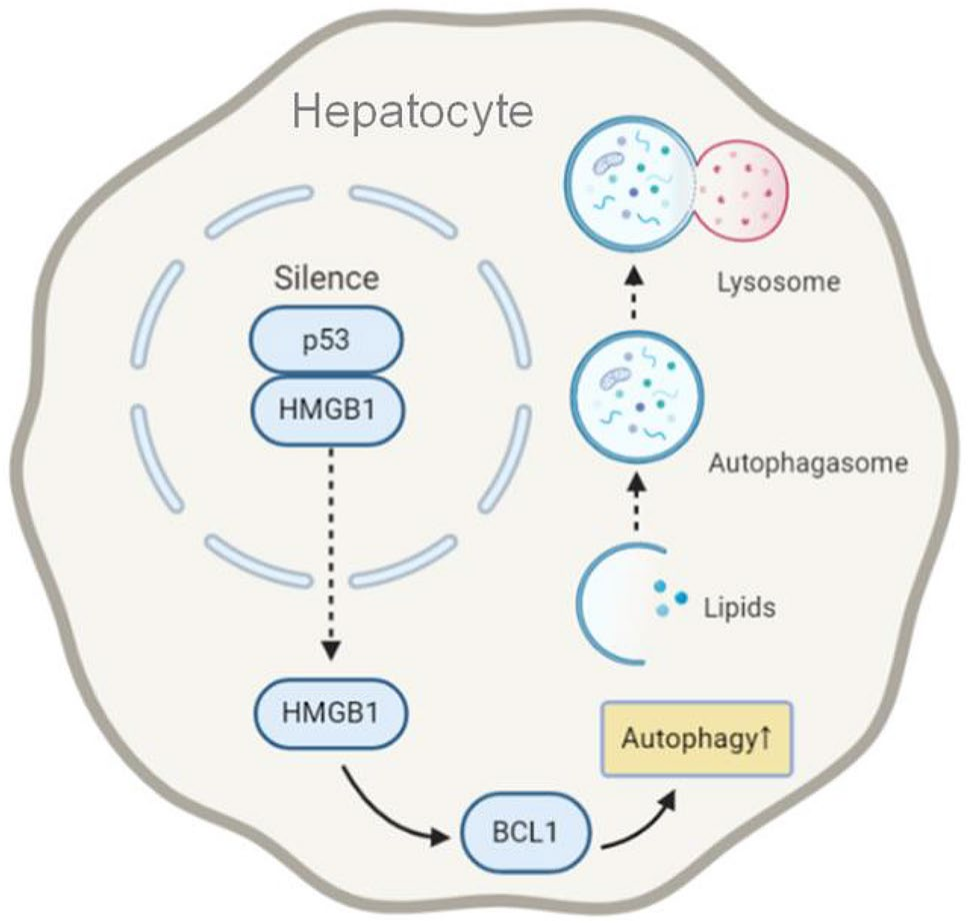

**Electronic supplementary material:**

The online version of this article (10.1007/s12072-020-10068-4) contains supplementary material, which is available to authorized users.

## Introduction

Nonalcoholic fatty liver disease (NAFLD) is a chronic liver disease commonly encountered in clinical practice in Western countries [1]. There has been a rise in the prevalence of NAFLD in developing countries as well, making NAFLD a more common liver disease worldwide and resulting in a growing global burden of disease [2]. NAFLD is a general term encompassing a spectrum of liver diseases from hepatosteatosis, steatohepatitis, steatohepatofibrosis to end-stage liver diseases, such as cirrhosis and hepatocellular carcinoma (HCC) [1]. Furthermore, it can aggravate glucose and lipid metabolic disorders, giving rise to diabetes, coronary heart disease and other metabolic diseases [3]. As the pathogenesis of NAFLD is complicated and remains to be further elucidated, there is still no pharmacological treatment with definite curative effects for NAFLD. Lifestyle intervention remains the most important treatment.

In recent years, the role of the tumor suppressor p53 in metabolic diseases has drawn increasing attention. The function of p53 has been studied for years in apoptosis, cell cycle arrest, cell senescence, differentiation and angiogenesis. Recent studies found that p53 also plays an important role in regulating energy metabolism [4–7]. It has been reported that the expression level of p53 in the adipose tissue of hereditary ob/ob obese mice is significantly higher than that of wild-type mice [8]. In addition, p53 can increase the accumulation of lipid droplets by regulating endoplasmic reticulum stress [9] and participate in the development of obesity by regulating adipose tissue differentiation [10]. In addition, p53 is involved in free fatty acid-induced pancreatic beta cell dysfunction and apoptosis [11]. Moreover, in a high-fat diet (HFD)-induced NAFLD mouse model, the p53 inhibitor attenuated weight gain, alanine aminotransferase levels, hepatosteatosis, oxidative stress and apoptosis compared to the control treatment [12]. Pharmacological stimulation of p53 ameliorated diet-induced nonalcoholic steatohepatitis [13]. Together, the results of these studies suggest a close relationship between p53 and NAFLD.

On the other hand, autophagy, which is a natural, regulated mechanism of cell growth, differentiation, survival and homeostasis in eukaryotic cells, also plays an important role in lipid metabolism. During starvation, autophagy pathways in cells are activated to maintain cellular energy metabolism [14]. Therefore, autophagy activation is a self-protective mechanism. Studies have shown that intracellular lipid droplets colocalize with autophagic lysosomes [11, 15], while intracellular lipid accumulation is significantly increased in autophagy-deficient cells compared with that in normal cells under the stimulation of fatty acids [15]. In addition, the activity of autophagy is altered during NAFLD, showing short-term activation and long-term inhibition [16]. Moreover, several compounds have been proven to be effective in alleviating NAFLD through autophagy activation [17–19].

Previous studies suggested a complex and dual relationship between p53 and autophagy that depended on the activation state, intracellular distribution and mutation of p53 [20]. P53 pathway has been found to regulate ATG genes such as LC3B and ATG7 [21]. On the one hand, p53 activation induced autophagy through multiple pathways via AMP-activated protein kinase (AMPK), damage-regulated autophagy modulator (DRAM) and Sestrin 2 [22–24]. Interestingly, on the other hand, loss of p53 also induced autophagy. In p53-functional HCT116 cells, p53 was found to bind to the HMGB1 protein in the nucleus, and silencing of p53 activated autophagy by promoting the expression of cytosolic HMGB1 and the autophagy modulator Beclin-1 (BCL1) [25]. Furthermore, the mutation of p53 also affected autophagy through additional regulatory mechanisms [26–28].

Collectively, these published studies suggested the involvement of the p53–autophagy axis in the pathogenesis of NAFLD, although its mechanism remains obscure. In this study, we focused on the effect of functional p53 silencing on hepatic autophagy and NAFLD as well as the associated mechanisms.

## Materials and methods

### Animals and HFD modeling

p53 heterozygous knockout mice (C57BL/6 background) were purchased from Biocytogen Co., Ltd. (Beijing, China). Animals were bred and maintained at 23 ± 2 °C with a 12-h light/12-h dark cycle at the Medical Science Institution of Zhejiang Province (Hangzhou, China). The p53 genotypes of the offspring mice were identified by the methods used by the facility (Supplementary Figure 1). Male adult wild-type (p53^+/+^) and knockout (p53^−/−^) mice weighing 19–23 g were randomly separated into two groups. For mice of both genotypes, the mice were randomly divided into two groups that were fed either a standard chow diet (SCD) provided by the Medical Science Institution of Zhejiang Province (Hangzhou, China) or a high-fat diet (D12492; Research Diets, New Brunswick, NJ, USA) for 8 weeks (*n *= 6). During the study, mice were given free access to water. The mice were killed, and the liver samples were separated and fixed in 10% neutral formalin or rapidly frozen in liquid nitrogen for further analysis. The fixed liver samples were embedded in paraffin, sectioned and then stained with hematoxylin and eosin (H&E) for histological examination according to the morphological criteria described previously [29]. The liver samples were analyzed with a transmission electron microscope (HT7700, Hitachi, Japan) after being fixed in 2.5% malondialdehyde. All institutional and national guidelines for the care and use of laboratory animals were followed.

### Glucose tolerance test (GTT) and insulin tolerance test (ITT)

For GTT, the mice were fasted for 16 h in advance in the 6th week of modeling. For ITT, the mice were fasted for 6 h in advance in the 7th week of modeling. A blank blood sample was taken from the tail end at time 0 to test the glycemic index using commercial test strips. The mice were then intraperitoneally injected with glucose (1 g/kg) for GTT or insulin (1 U/kg) for ITT, and blood samples were collected and tested to determine the glycemic index at 15, 30, 60, 90, and 120 min after injection. Feeding was resumed immediately after the experiment.

### Cell culture, PA modeling and drug treatment

HepG2 and Huh7 cells (American Type Culture Collection, Manassas, VA) were maintained in Dulbecco’s modified Eagle medium (DMEM) containing 10% fetal bovine serum (FBS) and 100 U penicillin/streptomycin under 5% CO_2_ at 37 °C. The cells were seeded overnight to allow them to adhere to the plate. PA was dissolved in phosphate-buffered saline (PBS) containing 25% BSA and added to the cell culture medium, followed by 24 h of incubation before harvest. For the evaluation of autophagic flux, the cells were treated with 10 μM chloroquine for 4 h.

### Cell transfection

Lipofectamine RNAiMAX reagent (Thermo Fisher Scientific, Waltham, MA USA) was used for siRNA transfection according to the manufacturer’s instructions. The siRNAs were synthesized by Sangon Biotech Co., Ltd. (Shanghai, China), and the cells were transfected with the siRNAs at a final concentration of 50 μM and maintained for 48 h until harvest. The sequences of the siRNAs are as follows: NC (forward: UUCUCCGAACGUGUCACGU dTdT, reverse: ACGUGACACGUUCGGAGAA dTdT); human p53 (forward: CACUACAACUACAUGUGUA dTdT, reverse: UACACAUGUAGUUGUAGUG dTdT); human HMGB1 (forward: CUGAUGCAGCUUAUACGAA dTdT, reverse: UUCGUAUAAGCUGCAUCAG dTdT); mouse p53 (forward: GUAAACGCUUCGAGAUGUU dTdT, reverse: AACAUCUCGAAGCGUUUAC dTdT); mouse Hmgb1 (forward: AUGCAGCUUAUACGAAGAUAA dTdT, reverse: UUAUCUUCGUAUAAGCUGCAU dTdT). Lipofectamine 3000 reagent (Thermo Fisher Scientific) was used for plasmid transfection, and 2 μg plasmids were transfected into each well (6-well plate). The pEGFP-LC3 plasmid was a generous gift from Professor Min Zheng (Zhejiang University, Hangzhou, China).

### Mouse primary hepatocyte isolation and culture

Hepatocytes from male p53 wild-type or null mice (8 weeks) were isolated using a two-step perfusion and collagenase digestion method as described previously [30]. The isolated cells were seeded on plates coated with rat tail collagen (Yisheng Biotechnology, Shanghai, China) and cultured in DMEM (supplemented with 10% FBS and 100 U penicillin/streptomycin) for the first 4 h, and then the medium was exchanged (DMEM/F12 without FBS) until harvest.

### Triglyceride (TG) determination

The hepatic and intracellular TG levels were analyzed using a commercial kit (E1013-105, Applygen Technologies, Beijing, China) according to the manufacturer’s instructions. Briefly, the liver tissues or collected cells were treated with lysis buffer on ice. The homogenates or lysates were incubated at 70 °C for 10 min and then centrifuged at 2000 rpm for 5 min at room temperature. The supernatant was assessed with the relevant working solution. The protein concentration in the resulting lysates was determined using a bicinchoninic acid protein assay kit (P1513, Applygen Technologies Inc.). The TG values were normalized according to the total protein levels.

### Oil red O (ORO) staining

Cryosections of liver tissues or cell slides were stained with oil red O according to a standard protocol (Nanjing Jiancheng Bioengineering Institute, Nanjing, China). The results were examined using a Leica DM5000B microscope (Leica, Heidelberg, Germany). The data shown were obtained from one representative experiment with three independent replicates.

### Immunochemistry

Paraffinized liver sections were deparaffinized with xylene and rehydrated, followed by incubation with 3% hydrogen peroxide. Heat epitope retrieval was performed in target retrieval solution at pH 7.5 for 20 min. The sections were preblocked with 10% goat serum (ZSGB-BIO, Beijing, China) and then incubated with HMGB1 antibody (Cell Signaling Technology, Beverly, MA, USA) at a dilution of 1:100 overnight at 4°C. Tissue sections were stained with HPR secondary antibody (dilution: 1:100, ZSGB-BIO) for 1 h at 37 °C in an incubator. Immunoreactivity was detected using a DAB kit (ZLI-9017, ZSGB-BIO, Beijing, China) and visualized as brown staining. Slides were counterstained with hematoxylin. The data shown were obtained from one representative experiment with three independent replicates.

### Immunofluorescent staining

Cells were seeded onto rat tail collagen-coated cover glasses (in a 24-well plate). The cell slides were fixed with 4% paraformaldehyde and incubated with 0.3% Triton X-100. Then, the cells were incubated in goat serum solution (ZSGB-BIO, Beijing, China) for 30 min at room temperature, followed by incubation with primary antibodies at 4 °C overnight and a secondary antibody (goat anti-rabbit IgG H&L Alexa Fluor 488/594 ab150077/ab150080, Abcam, Cambridge, UK) at room temperature for 1 h. DAPI staining (1 μg/ml, Thermo Fisher) was performed immediately before mounting. Images were captured with an Olympus IX81-FV1000 confocal microscope. For the liver slides, the first few steps were the same until the overnight primary antibody incubation was performed, and the subsequent steps were the same as those used for the cell slides described above. The data shown were obtained from one representative experiment with three independent replicates.

### Total RNA extraction and qPCR

Total RNA was extracted from the liver tissues or cultured cells using RNAiso Plus reagent according to the manufacturer’s instructions (TaKaRa Biotech, Kyoto, Japan), followed by random reverse transcription to cDNA using the PrimeScript RT reagent kit DRR036A (TaKaRa Biotech). Quantitative reverse-transcription polymerase chain reaction (PCR) analysis was performed using the SYBR Premix Ex Taq II kit RR820A (TaKaRa Biotech) on a CFX96 real-time PCR system (Bio-rad, Hercules, CA, USA). The glyceraldehyde-3-phosphate dehydrogenase gene was included with each run for the normalization expression. The gene-specific primer sequences were as follows (5′–3′): human *GAPDH* (forward: TCAACGACCACTTTGTCAAGCTCA, reverse: GCTGGTGGTCCAGGGGTCTTACT); human *p53* (forward: GAGGTTGGCTCTGACTGTACC, reverse: TCCGTCCCAGTAGATTACCAC); human *HMGB1* (forward: TATGGCAAAAGCGGACAAGG; reverse: CTTCGCAACATCACCAATGGA); mouse *Gapdh* (forward: AGGTCGGTGTGAACGGATTTG, reverse: GGGGTCGTTGATGGCAACA); mouse *p53* (forward: AGGTCGGTGTGAACGGATTTG, reverse: GGGGTCGTTGATGGCAACA); and mouse *Hmgb1* (forward: AGGTCGGTGTGAACGGATTTG, reverse: GGGGTCGTTGATGGCAACA).

### Protein extraction and western blot

Total proteins were extracted from liver tissues or cultured cells using RIPA assay lysis buffer (Applygen Technologies, Beijing, China) and quantified using the BCA Protein Assay (Beyotime, Jiangsu, China). Nuclear and cytoplasmic proteins were extracted using a commercial kit (P1201, Applygen Technologies, Beijing, China). Forty micrograms of protein extract was separated by 12% SDS-PAGE and electrophoretically transferred onto polyvinylidene fluoride membranes (0.2 μM, Millipore, MA). Then the membranes were blocked with 5% nonfat milk in Tris-buffered saline at room temperature and incubated at 4 °C overnight with primary antibodies, including GAPDH (#2118), ACTB (#4970), LC3 (#4108), Beclin1 (#3495), HMGB1 (#6893), and LaminB1 (#13435) from Cell Signaling Technology, p53(ab26) from Abcam, p21(556430) from BD (Franklin Lakes, NJ, USA), and P62 (#PM045) from MBL International (Woburn, MA, USA). Incubation with HRP-conjugated anti-rabbit or anti-mouse IgG secondary antibody (Dawen Biotec, Hangzhou, China) was performed for 1 h at room temperature. Specific bands were visualized using an ECL detection kit (P0018, Beyotime, Jiangsu, China) and photographed with a ChemiScope 6000 Pro Touch (Clinx Science Instruments, Shanghai, China). The data shown were obtained from one representative experiment with three independent replicates.

### Statistics

The data were expressed as the mean ± SEM and analyzed using GraphPad Prism 6 (GraphPad Software Inc., San Diego, CA). One-way ANOVA followed by Dunnett’s multiple comparison post hoc test or an unpaired Student’s *t* test was used for the statistical analysis. Only the comparisons indicated above the bars were performed, and differences were considered significant if the probability (*p* value) was less than 0.05 (*p* < 0.05).

## Results

### Autophagy is induced in p53-null mouse livers

**Fig. 1:**
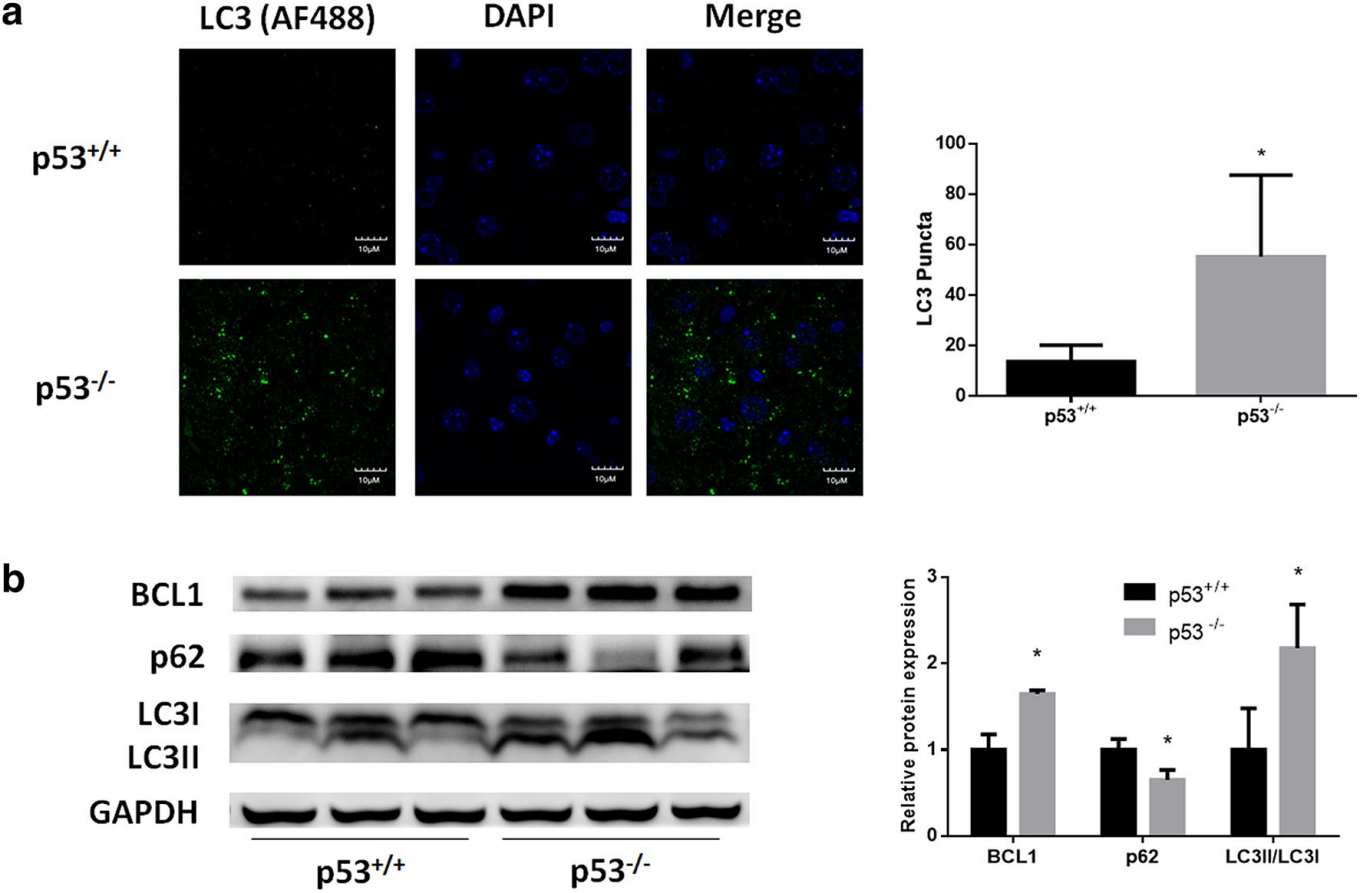
Induction of autophagy by p53 knockout in mouse livers. **a** Confocal microscopy analysis of autophagosomes by immunofluorescent staining of LC3 (AF488) in p53^+/+^ and p53^−/−^ mouse liver slides. **b** Western blot analysis for autophagy determination in p53^+/+^ and p53^−/−^ mouse livers. DAPI was chosen for nuclear staining. *p < 0.05, compared with p53^+/+^ mice

To investigate the effect of p53 loss on autophagy in hepatocytes, we performed immunofluorescent staining of mouse liver sections. As indicated by LC3 immunostaining, the number of punctate autophagosomes was significantly increased in p53-null mouse livers (Fig. 1a). Western blotting analysis revealed an increase in the LC3II/LC3I ratio, as well as a decrease in the p62 expression in p53-null mouse livers, which demonstrated the upregulation of autophagic flux (Fig. 1b). Furthermore, we found that p53 loss-induced autophagy was associated with an increase in BCL1 protein expression (Fig. 1b).

### Autophagy is induced in HepG2 cells with functional p53 silencing but not in Huh7 cells with p53 silencing

**Fig. 2:**
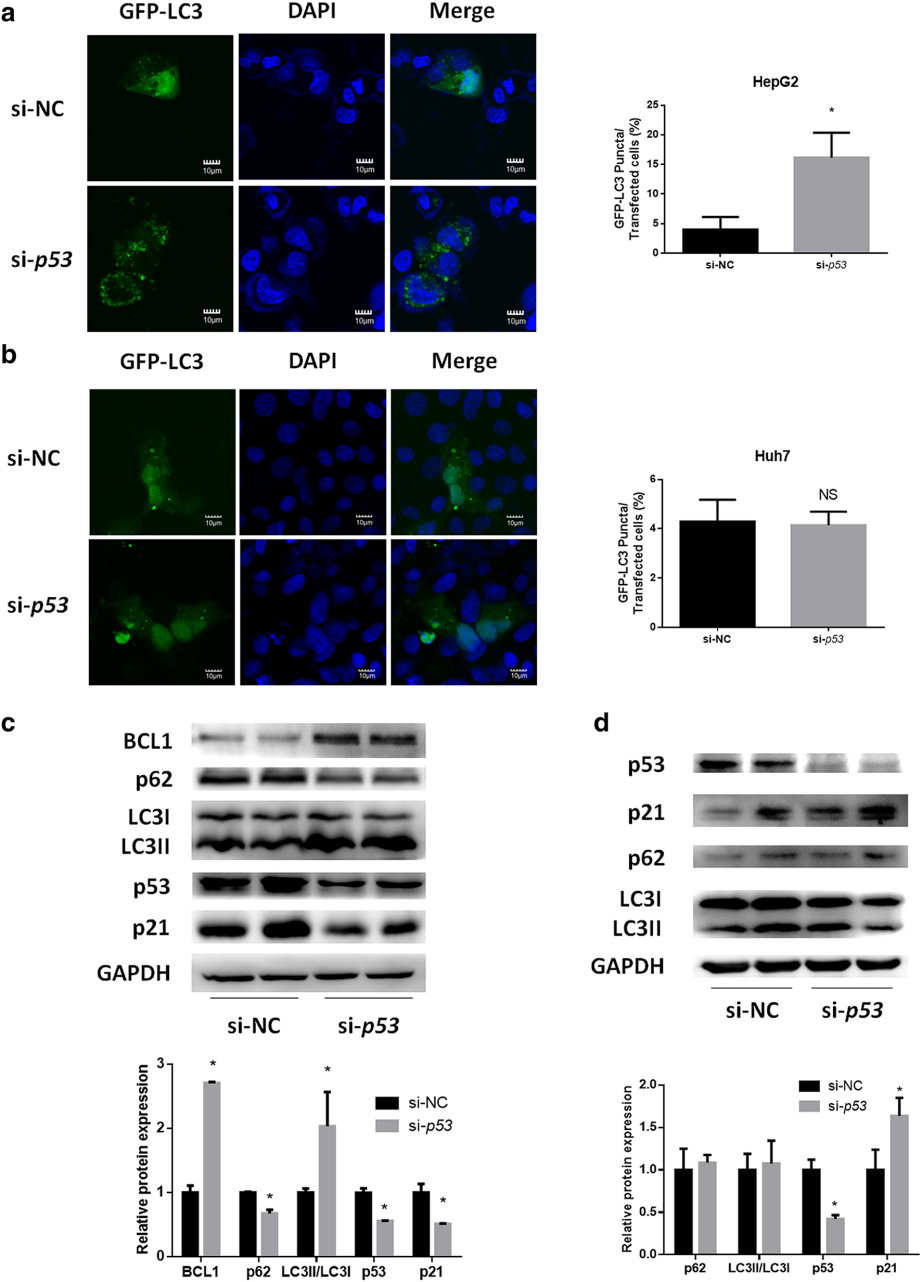
Effect of p53 silencing on autophagy in vitro. **a, b** Confocal microscopy analysis of autophagosomes in **a** HepG2 and **b** Huh7 cells. Cells were first transfected with p53 siRNA for 24 h, followed by pEGFP-LC3 plasmid transfection for another 24 h. DAPI was chosen for nuclear staining. **c, d** Western blotting analysis of autophagy- and p53-related pathways in p53-silenced **c** HepG2 and **d** Huh7 cells. *p < 0.05, compared with the si-NC group

We next investigated how autophagy was regulated when p53 was silenced in human liver-derived HepG2 and Huh7 cells. We first confirmed p53 function in these two cell lines by measuring the mRNA level of the p53 target gene p21 after treating cells with the p53 activator Nutlin3a and inhibitor PFTa. As illustrated in Supplementary Figure 2a–d, nutlin3a treatment time-dependently induced p21 transcription in HepG2 cells, while the same treatment did not induce p21 in Huh7 cells. Furthermore, PFTα treatment displayed a rapid and significant inhibition effect on p21. However, no such inhibition effect was observed in Huh7 cells. These results suggest that p53 is functional in HepG2 cells, but not in Huh7 cells. Next, cells were transfected with siRNA to knockdown p53 mRNA levels with a silencing efficiency of 91.4% for HepG2 cells and 89.3% for Huh7 cells (Supplementary Figure 3a, b). Confocal analysis showed that p53 knockdown increased the number of punctate autophagosomes in HepG2 cells (Fig. 2a). In contrast, knockdown of p53 did not increase the number of punctate autophagosomes in green fluorescent protein (GFP)-LC3-transfected Huh7 cells (Fig. 2b). A protein expression assay also found increased p62 degradation, an increased LC3II/LC3I ratio and an increased level of BCL1 expression in HepG2 cells with p53 silencing (Fig. 2c). However, in Huh7 cells, p53 knockdown did not affect p62 expression or the LC3II/LC3I ratio (Fig. 2d). In addition, we observed an increase in the LC3II/LC3I ratio in cells with p53 silencing followed by chloroquine (a well-known autophagy inhibitor) treatment versus that in cells treated with vehicle alone, indicating that p53 silencing indeed induced autophagic flux (Supplementary Figure 4).

### Autophagy contributes to hepatic lipid degradation

To investigate the association between autophagy and lipid degradation in hepatocytes, we performed colocalization analysis of autophagosomes and lipids. In PA-treated HepG2 cells, immunolabelled LC3 was found to colocalize with BODIPY 493-labeled lipids (Supplementary Figure 5a). Furthermore, when the cells were treated with chloroquine, the intracellular TG level was significantly elevated in the absence of PA (Supplementary Figure 5b), which was then confirmed by ORO staining (Supplementary Figure 5c). These results suggested a strong association between autophagosomes and intracellular lipids in the liver.

### Symptoms of NAFLD are attenuated in p53-null HFD-fed mice

**Fig. 3:**
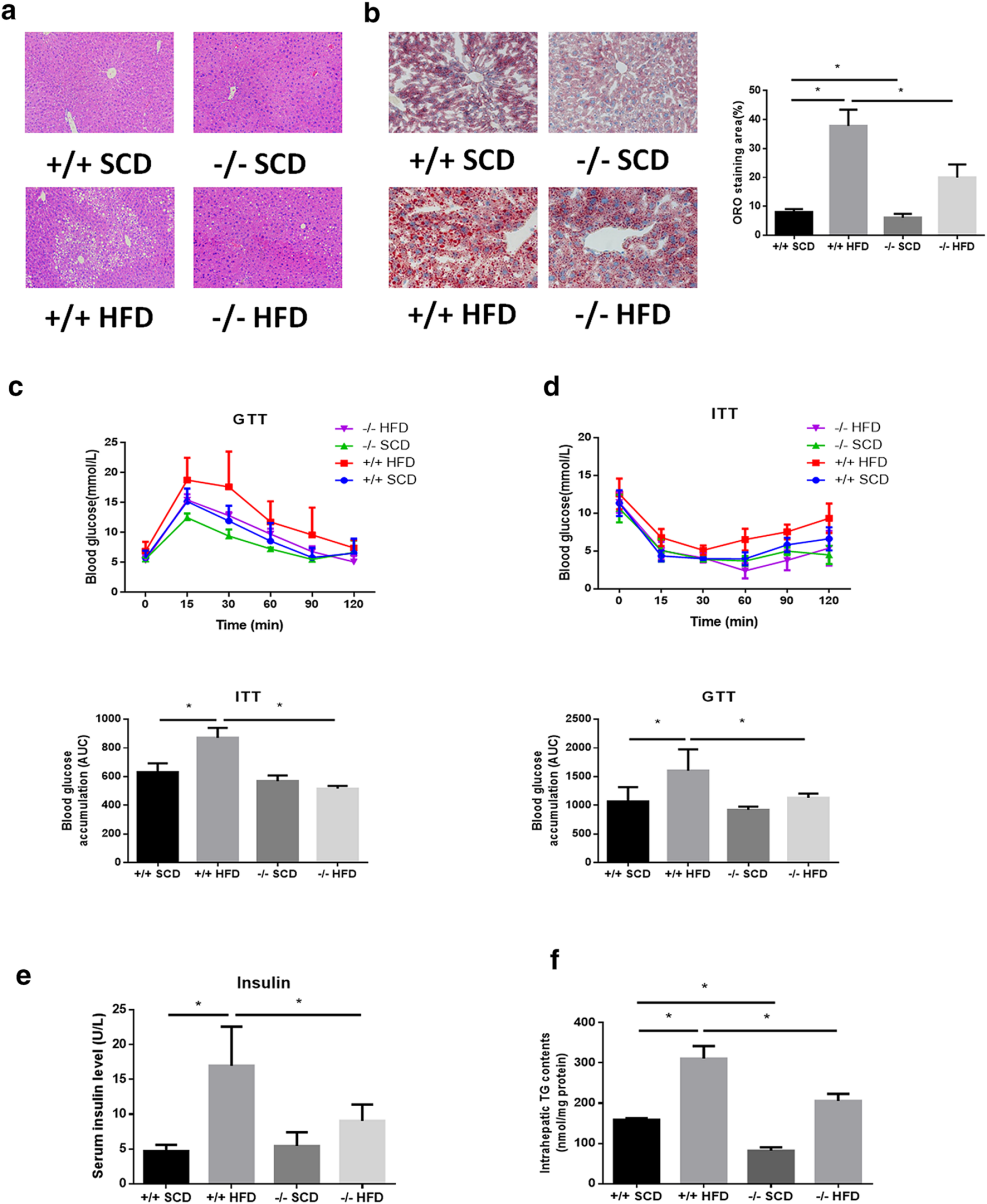
Attenuated NAFLD symptoms in HFD-fed p53-null mice. **a** H&E staining and **b** ORO staining of livers (× 200). **c** Glucose tolerance test of mice after HFD modeling for 6 weeks. **d** Insulin tolerance test of mice after HFD modeling for 7 weeks. **e** Triglyceride levels in mouse livers. *p < 0.05

To explore the effect of p53 loss on HFD-induced NAFLD in mice, male p53 WT and null mice were fed either a SCD or HFD for 8 weeks. No significant difference in food or water intake was observed in any of the animal groups (data not shown). H&E staining as well as ORO staining suggested obvious hepatic steatosis in mice fed a HFD compared to mice fed a SCD (Fig. 3a, b). Additionally, ITT and GTT indicated impaired insulin sensitivity in mice fed a HFD (Fig. 3c, d). Meanwhile, mice fed a HFD exhibited higher serum insulin level (Fig. 3e). Furthermore, HFD feeding also significantly elevated hepatic TG levels (Fig. 3f). Interestingly, in HFD-fed mice, p53-null mice exhibited the attenuation of hepatic steatosis and lipid and TG accumulation as well as lower insulin and improved ITT/GTT performance compared with wild-type mice (Fig. 3a–e). These results suggest a protective effect of p53 loss on NAFLD in vivo.

### NAFLD symptoms are alleviated in PA-treated primary hepatocytes and HepG2 cells but not in Huh7 cells with p53 silencing

**Fig. 4:**
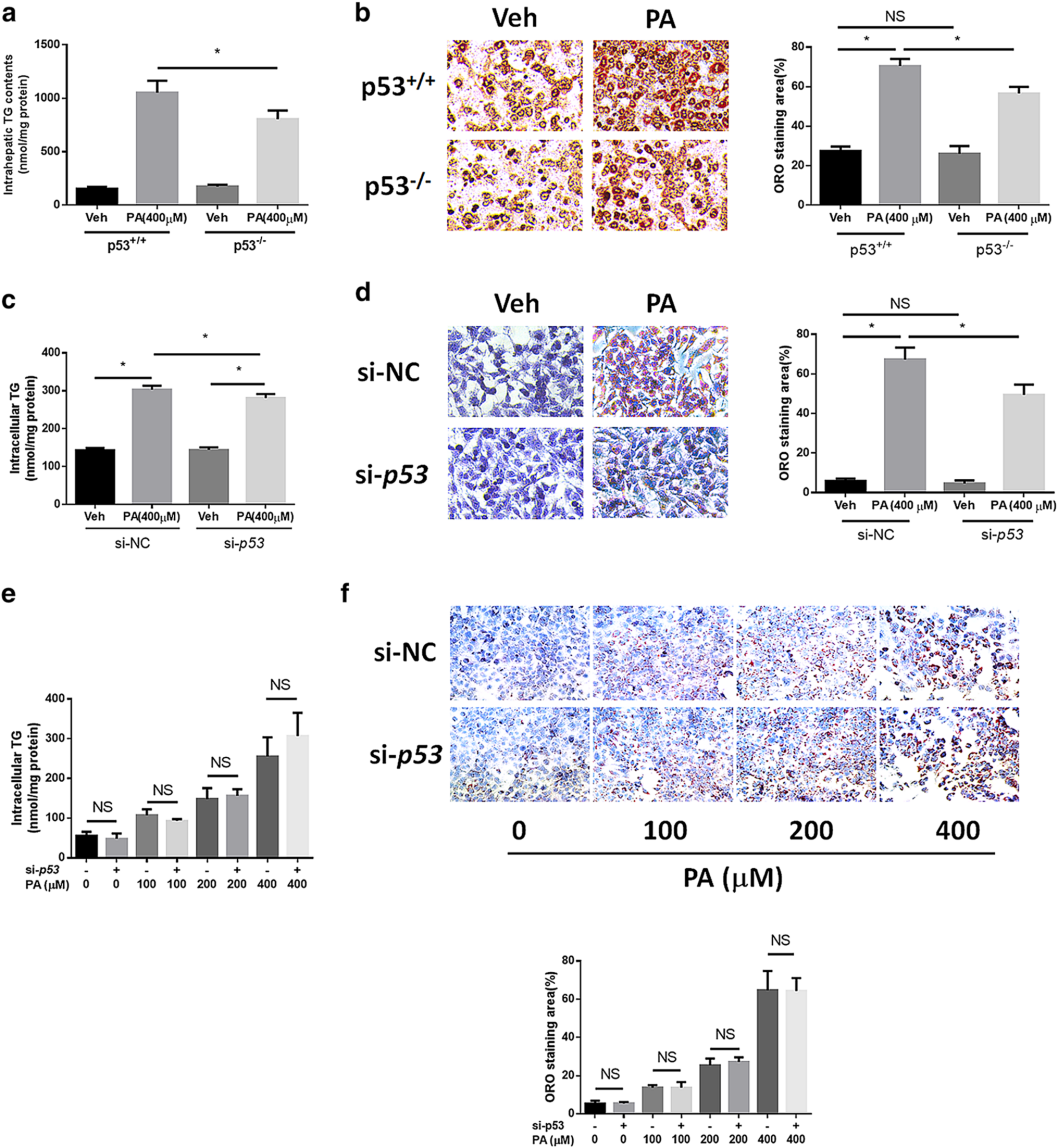
p53 silencing ameliorates NAFLD symptoms in vitro. **a** Intracellular triglyceride levels and **b** ORO staining of PA-treated primary hepatocytes from p53^+/+^ and p53^−/−^ mice (× 200). **c** Intracellular triglyceride levels and **d** ORO staining of p53-silenced HepG2 cells treated with PA (× 200). **e** Intracellular triglyceride levels and **f** ORO staining of p53-silenced Huh7 cells treated with different concentrations of PA for 24 h (× 100). *p < 0.05; *NS* no significance

We confirmed the protective effect of the knockdown of functional p53 on NAFLD in vitro. Firstly, we confirmed the function or dysfunction of p53 by detecting the induction effect of p53 agonist on p21 transcription in primary hepatocytes isolated from wild type or knockout mice (supplementary Figure 2e, f). We established a NAFLD model in mouse primary hepatocytes via 400 μM PA treatment for 24 h. Elevated intracellular TG levels and obvious lipid staining demonstrated steatosis in both cell lines (Fig. 4a, b). In hepatocytes from p53-null mice, reduced TG levels and attenuated lipid staining were observed. Similarly, 400 μM PA treatment for 24 h also resulted in obvious TG level elevation and strong lipid staining, while p53 knockdown resulted in reduced TG levels and attenuated lipid staining (Fig. 4c, d). However, p53 silencing had no significant effect on steatosis in Huh7 cells at multiple PA concentrations (Fig. 4e, f). Interestingly, p53 silencing exacerbated TG accumulation caused by 400 μM PA treatment in Huh7 cells.

### Hepatic HMGB1 translocation by p53 silencing

**Fig. 5:**
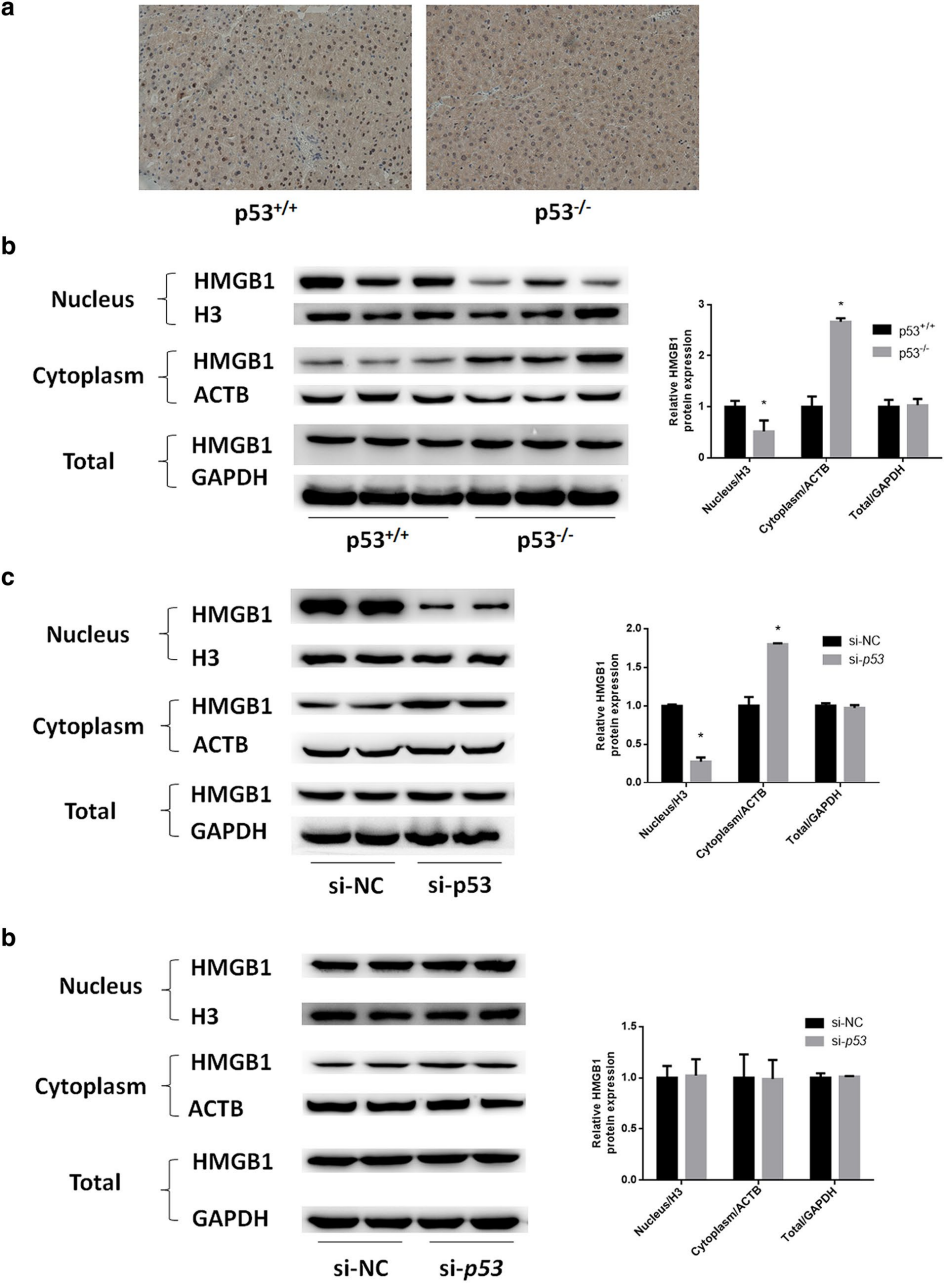
Hepatic HMGB1 translocation induced by p53 silencing. **a** Immunochemical analysis of HMGB1 expression in livers from p53^+/+^ and p53^−/−^ mice. **b** Western blotting determination of nuclear, cytoplasmic and total HMGB1 expression in p53^+/+^ and p53^−/−^ mouse livers. **c, d** Western blotting determination of nuclear, cytoplasmic and total HMGB1 expression in p53-silenced **c** HepG2 and **d** Huh7 cells. H3, ACTB and GAPDH were chosen as internal controls for nuclear, cytoplasmic and total protein, respectively. *p < 0.05, compared with the p53^+/+^ or si-NC group

HMGB1 is an autophagy regulator that activates BCL1 in the cytoplasm. We examined the distribution of HMGB1 in mouse livers via immunohistochemical analysis. Interestingly, HMGB1 existed mainly in the nucleus zone in hepatocytes derived from wild-type mice, but was more highly distributed in the cytoplasm in hepatocytes from p53-null mice (Fig. 5a). Additionally, the nuclear, cytoplasmic and total HMGB1 protein expression in mouse livers as well as in HepG2 and Huh7 cells was determined. As a result, in hepatocytes from p53-null mice and in p53-silenced HepG2 cells, HMGB1 expression was decreased in the nucleus but elevated in the cytoplasm, while no significant change was observed in the total level (Fig. 5b, c). Such phenomena were not observed in p53-silenced Huh7 cells (Fig. 5d). These results indicated that loss/silencing of functional p53 could cause the translocation of HMGB1 from the nucleus to the cytoplasm.

### HMGB1 knockdown blocks the induction of autophagy and the attenuating effect of p53 silencing on TG accumulation in HepG2 and primary hepatocytes

**Fig. 6:**
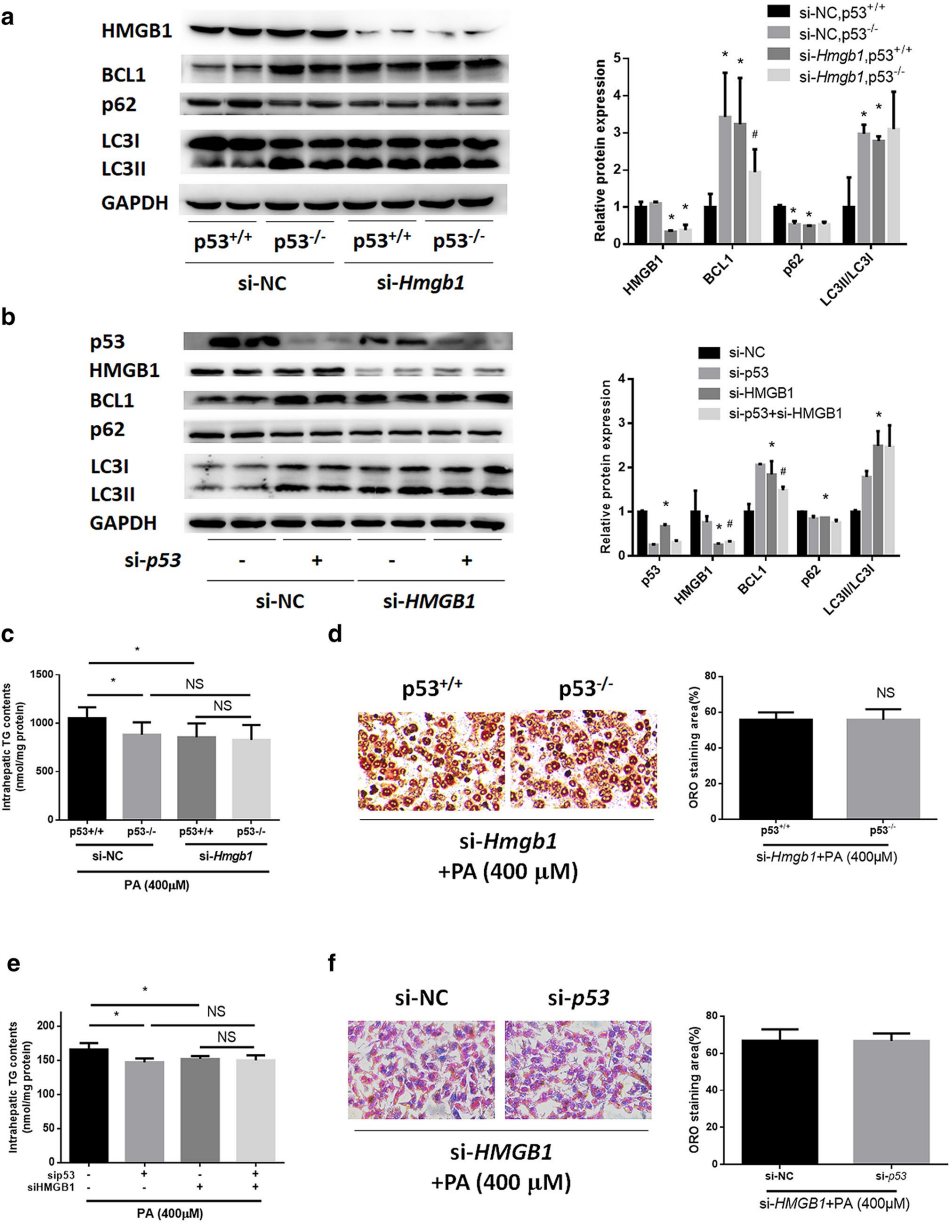
HMGB1 knockdown blocks the induction of autophagy as well as the attenuation of TG accumulation by p53 silencing in HepG2 and primary hepatocytes. **a** Western blotting results for *Hmgb1*-silenced primary hepatocytes from p53^+/+^ and p53^−/−^ mice. **b** Western blotting results for *HMGB1*- and *p53*-silenced HepG2 cells. **c, d** Intracellular triglyceride levels and ORO staining in *Hmgb1-*silenced primary hepatocytes from p53^+/+^ and p53^−/−^ mice with PA treatment for 24 h. **e, f** Intracellular triglyceride levels and ORO staining in *HMGB1-* and/or *p53*-silenced HepG2 cells treated with PA for 24 h. *p < 0.05, compared with the si-NC group; ^#^*p* < 0.05, compared with the si-*Hmgb1*/si-*HMGB1* group; *NS* no significance

To further investigate how HMGB1 participates in the regulatory mechanism of p53 in autophagy and NAFLD, we knocked down HMGB1 expression in HepG2 cells and primary hepatocytes with siRNAs with silencing efficiencies of 77.3% and 70.3%, respectively (Supplementary Figure 3d, e). In both cell lines, HMGB1 silencing counteracted the elevation of the LC3II/LC3I ratio, the upregulation of BCL1, and the degradation of p62 induced by p53 silencing (Fig. 6a, b). What’s more, we found upregulation of BCL1, inhibition of p62 and increase of LC3II/I ratio, following HMGB1 knocking down both in primary hepatocytes and HepG2 cells (Fig. 6a, b). Furthermore, we tested the effect of HMGB1 knockdown on PA-induced steatosis in p53-silenced cells. In HepG2 and primary hepatocytes from wild-type mice, HMGB1 silencing attenuated TG and lipid accumulation induced by PA administration (Fig. 6c–f). Interestingly, in both cell lines, HMGB1 knockdown offset the alleviating effect of p53 silencing on hepatic TG accumulation and steatosis. These results collectively suggested that HMGB1 contributed to the protective effect of p53 silencing on NAFLD by regulating autophagy. Furthermore, we also silenced p53 by siRNA transfection in primary hepatocytes from wild-type mice with a silencing efficiency of 92.5% (Supplementary Figure 3c) and found that *Hmgb1* knockdown also blocked autophagy induction and alleviated NAFLD symptoms via p53 knockdown (Supplementary Figure 6a–c).

## Discussion

Here, we identified and characterized a novel HMGB1-mediated autophagy pathway involved in the regulatory mechanism of p53 in NAFLD. We investigated the effect of functional p53 silencing on hepatic autophagy and NAFLD in vitro and in vivo and demonstrated the association between hepatic autophagy and lipid accumulation. We found that HMGB1 is a key protein mediating p53 function and regulating autophagy. The ameliorating effect of p53 silencing on NAFLD and its inductive effect on autophagy were counteracted when HMGB1 was knocked down, as we demonstrated in both primary hepatocytes and HepG2 cells. Due to limited resources, we did not utilize double knockout mice to investigate the role of HMGB1. Interestingly, in the livers of mice without HFD modeling, p53-null mice exhibited decreased TG levels and reduced lipid accumulation, indicating that p53 loss could alter physiological hepatic lipid metabolism.

Mutation of p53, a well-known tumor suppressor, in humans and its loss in mice contribute to tumorigenesis. In our study, HepG2 cells present a WT p53, whereas Huh7 are cells with a mutant p53 gene (C220T, https://p53.free.fr/). Interestingly, our study demonstrated that silencing of functional p53 could ameliorate NAFLD, a metabolic disease, which indicated a close but complicated link between lipid metabolism and cancer. In general, p53 mutation leads to the alteration of the normal biological function of p53, mainly in anti-tumor functions. Importantly, the mutation R172H of p53 can affect hepatosteatosis [31]. Similarly, in our study, the mutant p53 in Huh7 cells also exhibited different regulation pattern from normal p53, which was probably caused by the impaired binding of mutated p53 to HMGB1. As a result, p53 knockdown resulted in neither the intracellular translocation of HMGB1 nor induced autophagy in Huh7 cells. These results suggested that p53 mutation should be considered a strategy to exploit p53 silencing in clinical practice to combat NAFLD.

The interaction of p53 and HMGB1 was first discovered in colon-derived HCT116 cells [25] and confirmed in the current study to also occur in liver-derived cells. We found that p53 knockout/knockdown caused HMGB1 to translocate from the nucleus to the cytoplasm and to induce BCL1 expression, eventually promoting autophagy.

Interestingly, the signaling molecule HMGB1 has been demonstrated to be a “dangerous factor” in the pathogenesis of NAFLD. HMGB1 expression is elevated in NAFLD mouse livers, and inhibition of HMGB1 protects against NAFLD [32, 33]. In our study, we also observed the protective effect of HMGB1 knockdown on PA-induced TG elevation in primary hepatocytes and HepG2 cells. However, the reported mechanism of the function of HMGB1 in NAFLD is associated with the TLR4 pathway [34]. Thus, the role that HMGB1 plays in the development of NAFLD through the autophagy pathway has not yet been reported. In this study, we observed alternative expression of p62 and LC3II following HMGB1 knockdown in primary hepatocytes and HepG2 cells, strongly suggesting a potential novel mechanism involving HMGB1 in NAFLD that requires further investigation.

In addition, degradation of p62 and an increase in LC3II/LC3I expression were found in both the livers of HFD-fed wild-type mice and PA-treated HepG2 cells in our study (Supplementary Figure 7a, b), in accordance with the reported promotion of autophagy in a short-term NAFLD model [16, 35]. Considering the protective role of autophagy against NAFLD, activation of autophagy can be a compensatory mechanism for hepatocytes to process excess intracellular lipids. P53 silencing upregulates autophagy at the basal level to accelerate the disposal of excess lipids, and the activation of autophagy is a process secondary to HFD modeling. A similar situation also applies to HepG2 cells. On the other hand, in wild-type mouse livers and HepG2 cells, NAFLD modeling resulted in p53 activation (Supplementary Figure 7a, b), in accordance with previous studies [36–38]. Scores of studies have demonstrated that multiple pathways are involved in p53 activation to induce autophagy, such as the AMPK/mTOR, DRAM and SESEN2 pathways. Interestingly, p53 activation could also lead to autophagy arrest [39]. Therefore, the effect of activated p53 on autophagy during NAFLD can be complicated. We cannot identify whether the activation of autophagy by NAFLD modeling is a result of p53 activation or just a compensatory process. Therefore, the functional mechanism of p53 activation in NAFLD requires further research. What’s more, a recently published study proves the reciprocal regulation of autophagy on p53 [40]. So it is also interesting to further discover this possible regulation pattern in NAFLD.

In summary, we have demonstrated that the functional silencing of p53 plays an important role in protecting against NAFLD by activating autophagy in vivo and in vitro via the HMGB1 pathway. These findings are especially noteworthy for developing a NAFLD therapeutic strategy utilizing the pharmacological inhibition of p53. Since there is still no drug therapy for NAFLD, understanding the mechanisms can provide insight useful for identifying therapeutic targets and developing drugs for the treatment of NAFLD.

## Electronic supplementary material

Below is the link to the electronic supplementary material.

Supplementary file1 (DOCX 1354 kb)

## Abbreviations

NAFLD: Nonalcoholic fatty liver disease
PA: Palmitate
HMGB1: High mobility group box 1
HCC: Hepatocellular carcinoma
HFD: High-fat diet
AMPK: AMP-activated protein kinase
DRAM: Damage-regulated autophagy modulator
BCL1: Beclin-1
SCD: Standard chow diet
H&E: Hematoxylin and eosin
GTT: Glucose tolerance test
ITT: Insulin tolerance test
DEME: Dulbecco’s modified Eagle medium
FBS: Fetal bovine serum
PBS: Palmitate
GFP: Green fluorescent protein
CQ: Chloroquine
