# Supplementary material for: Silencing of functional p53 attenuates NAFLD by promoting HMGB1-related autophagy induction
Source: Hepatol Int. 2020 Jun 30;14(5):828–41. doi: 10.1007/s12072-020-10068-4 (PMC7561543; doi:10.1007/s12072-020-10068-4)
Supplement: Supplementary file 1 — Supplementary file1 (DOCX 1354 kb) [file 12072_2020_10068_MOESM1_ESM.docx]

Silencing of functional p53 attenuates NAFLD by promoting HMGB1-related autophagy induction

**Xuequn Zhang, Yiming Lin, Sisi Lin, Chunxiao Li, Jianguo Gao, Zemin Feng, Jinghua Wang, Jie Zhang, Hong Zhang, Yuwei Zhang, Xueyang Chen, Shenghui Chen, Chengfu Xu, Youming Li, Chaohui Yu, Hang Zeng**

**SUPPLEMENTARY INFORMATION**

**SI Figures**

**
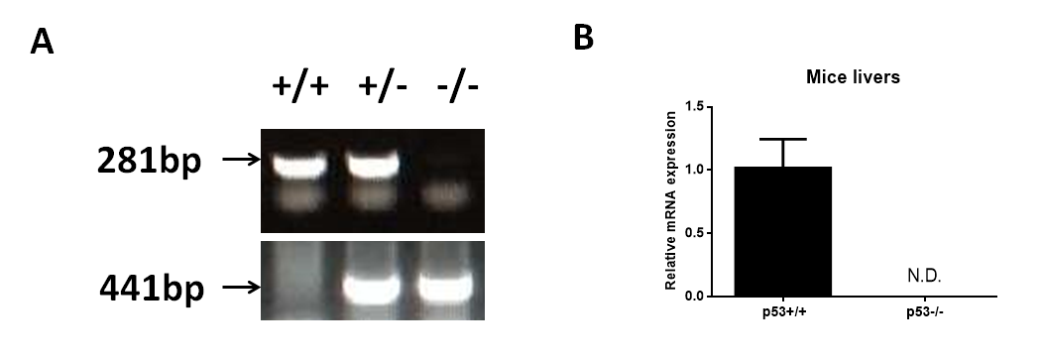
**

**Supplementary Figure. 1.** P53-null mice identification. (A) Agarose electrophoresis analysis for mice genomic DNA PCR products. (B) RT-qPCR analysis for p53 mRNA determination in mice livers. N.D. not detected.


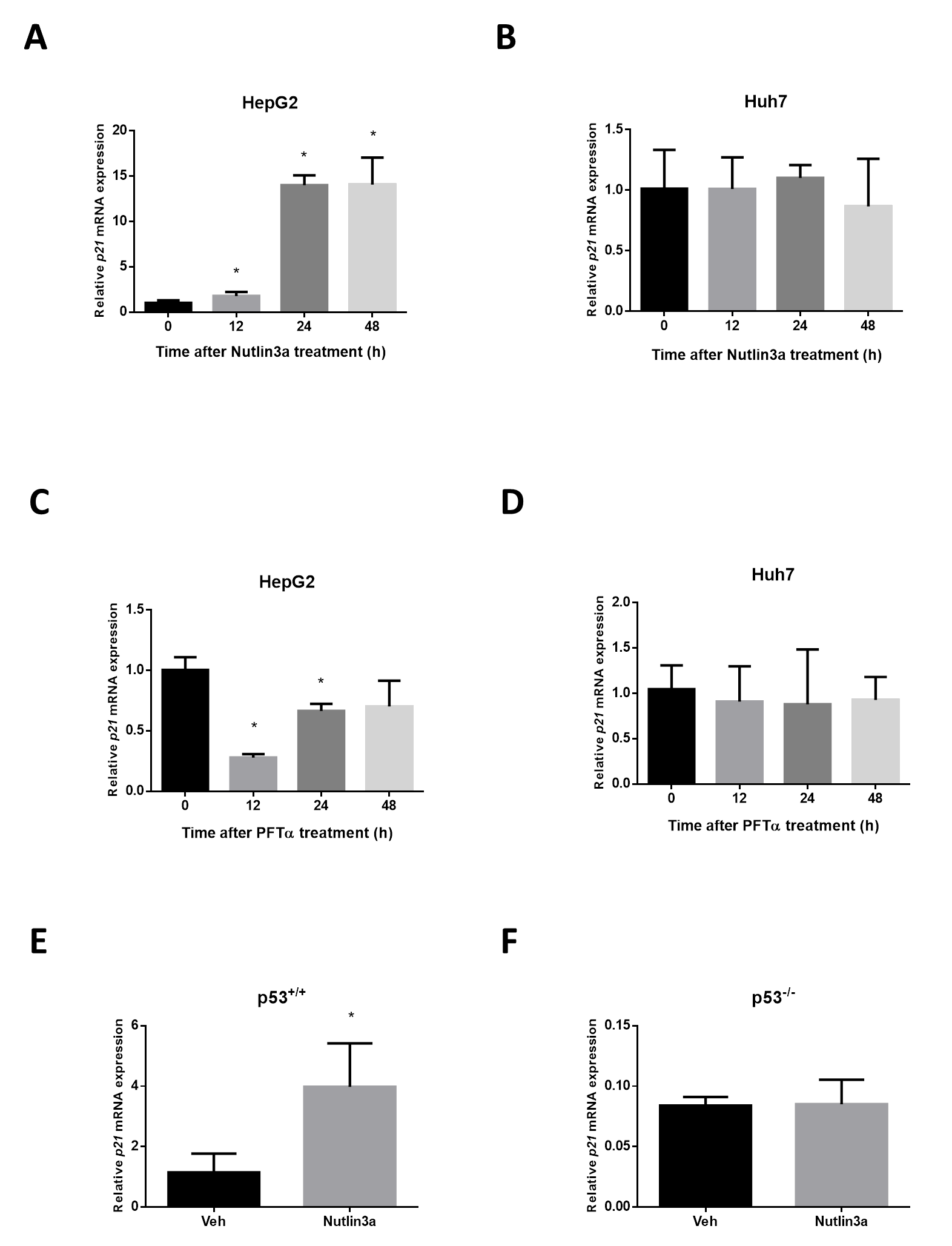


**Supplementary Figure. 2.** Confirmation of functional or dysfunctional p53 in HepG2, Huh7 cells and mouse primary hepatocytes. HepG2 and Huh7 cells were treated with (A, B) p53 activator Nutlin3a (10 μM) or (C, D) p53 inhibitor PFTα (50 μM) for 12, 24 and 48h. (E) Mouse primary hepatocytes isolated from p53^+/+^ or p53^-/-^ mice were treated with 10 μM Nutlin3a for 24 h. RT-qPCR analysis was performed to determinate p21 mRNA levels. *P<0.05, compared with 0 h or Veh, vehicle.


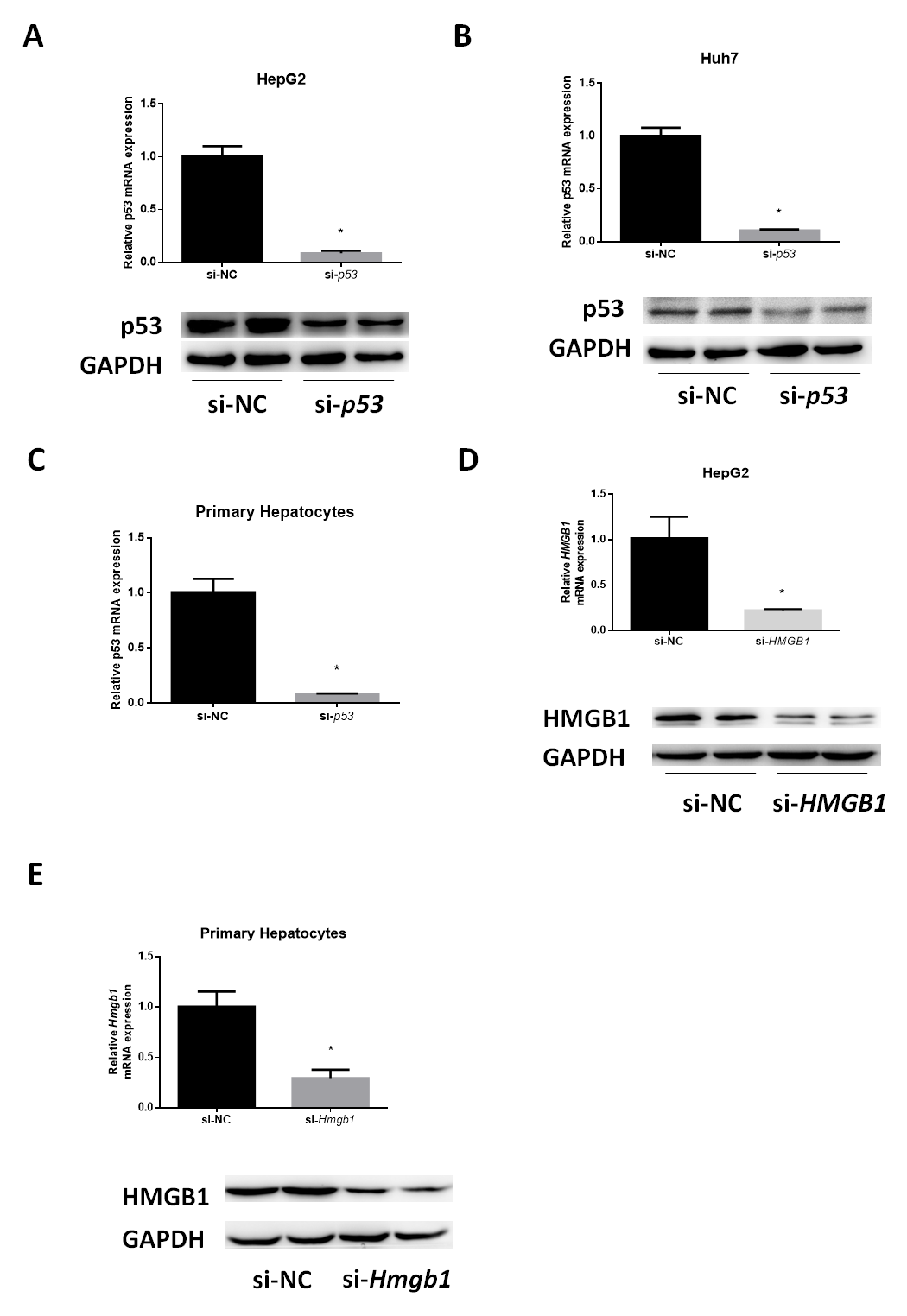


**Supplementary Figure. 3**. Silencing efficiency of siRNA transfection detected by RT-qPCR or Western blot in (A, B) human p53, (C) mouse p53, (D, E) Human and mouse HMGB1. *P<0.05.


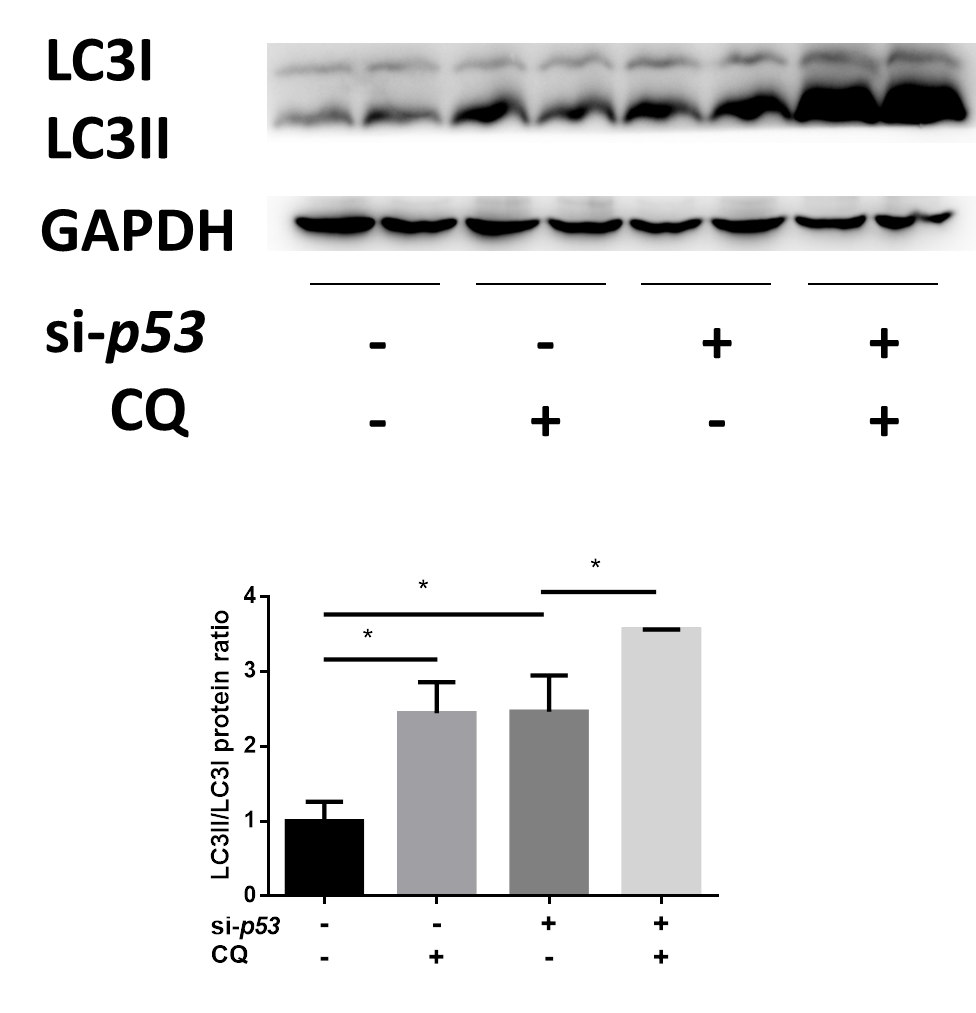


**Supplementary Figure. 4**. P53 silence promotes autophagic efflux. HepG2 cells were trancsfected with p53 siRNA for 24 hours, followed by 4-hour treatment with 10 μM of lysosomal inhibitor CQ. Increase in LC3II/LC3I protein ratio of p53-silenced cells after CQ treatment versus cells trancsfected with siRNA control confirms increase in autophagic flux. *P<0.05.


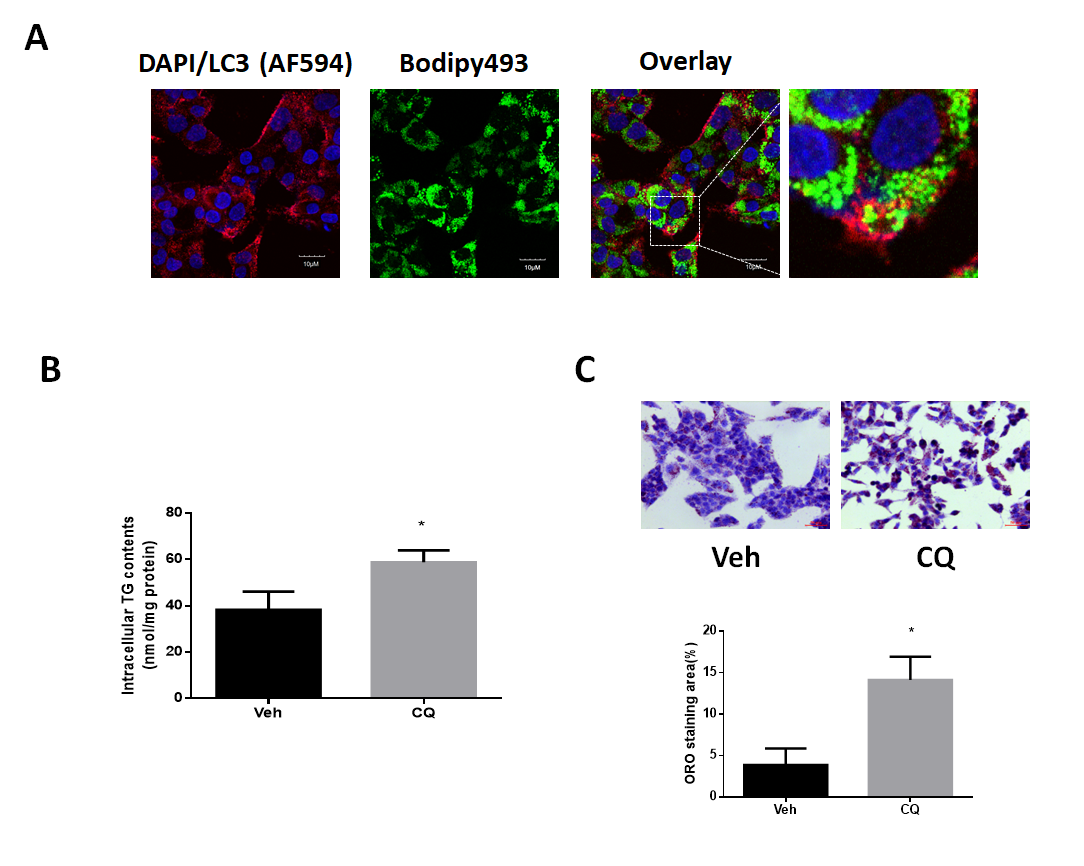


**Supplementary Figure. 5.** The association between autophagy and lipid degradation. (A) Autophagasomes indicated as LC3 staining (AF594) co-localized with lipids stained with bodipy493 in HepG2 cells by confocal analysis. Yellow area stands for overlay by green and red. (B, C) Intracellular triglyceride levels and oil red o staining of HepG2 cells treated with 50μM CQ for 24h. *P<0.05; Veh, vehicle.

**
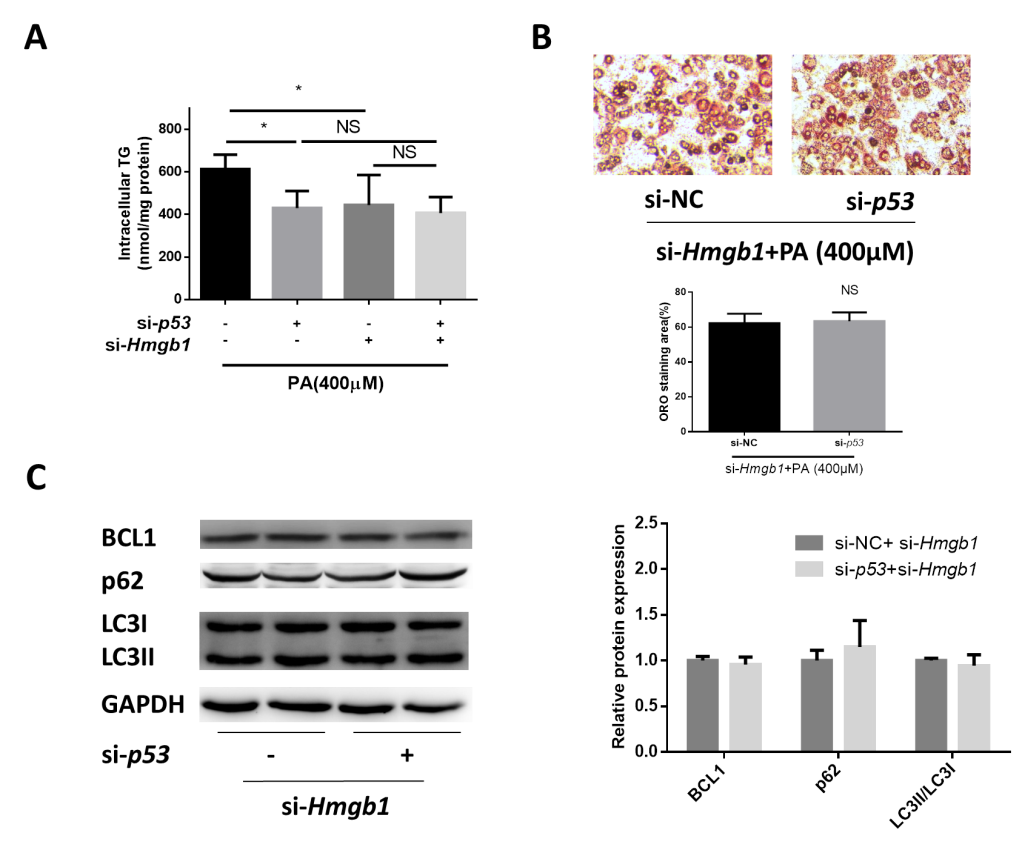
**

**Supplementary Figure. 6.** Hmgb1 silencing displayed no alleviating effect on TG or lipid accumulation and no induction of autophagy in p53-silenced wild type primary hepatocytes. (A, B) Intracellular triglyceride levels and oil red O staining results. The cells were transfected with siRNA for 48h, followed by PA incubation for another 24h. (C) Western blot analysis for autophagy-related proteins. The cells were transfected with siRNA for 48h. *P<0.05, compared with si-NC; NS, no significance.


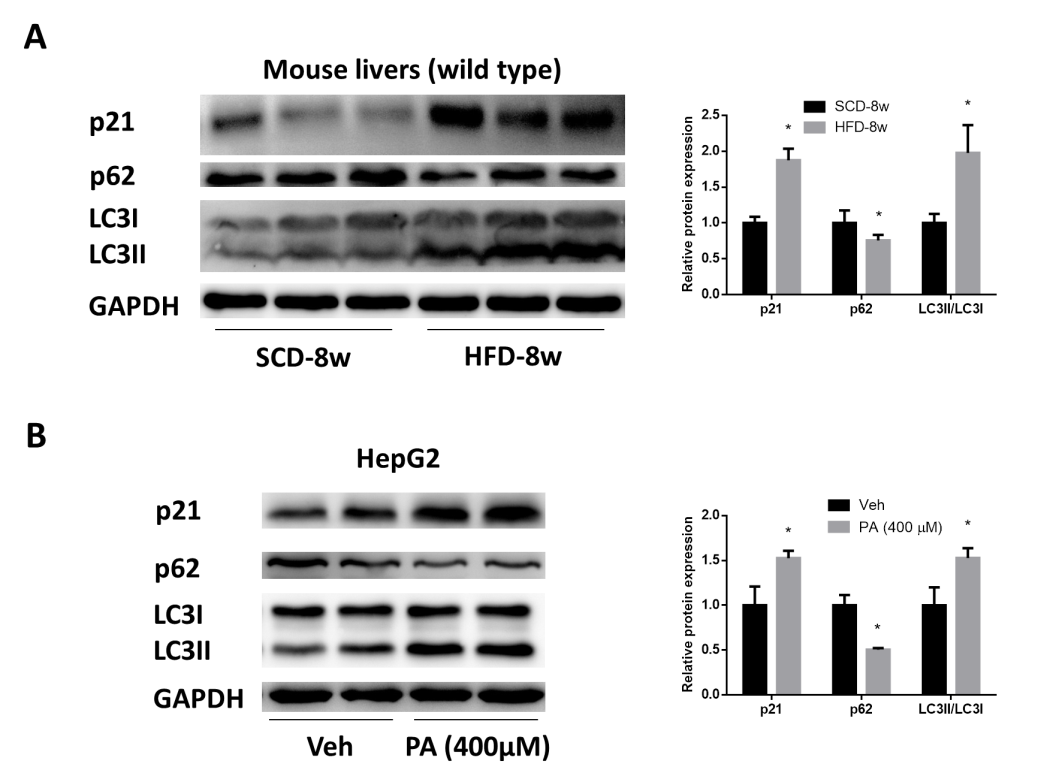


**Supplementary Figure. 7.** Autophagy and p53 were activated by NAFLD modeling. Western blot analysis for p62 and LC3 expression in (A) 8-week HFD-fed mice livers and (B) PA-treated HepG2 cells. *P<0.05, compared with SCD or vehicle group; Veh, vehicle.
